# Paliperidone Induced Hypoglycemia by Increasing Insulin Secretion

**DOI:** 10.1155/2016/1805414

**Published:** 2016-07-10

**Authors:** Tsubasa Omi, Keisen Riku, Motoyuki Fukumoto, Koji Kanai, Yumi Omura, Hiromune Takada, Hidenori Matunaga

**Affiliations:** Department of Psychiatry, Osaka General Medical Center, 3-1-56 Bandai-higashi, Sumiyoshi, Osaka 5588558, Japan

## Abstract

We report the case of a 41-year-old woman with schizophrenia who developed persistent hypoglycemia following paliperidone administration. After discontinuing paliperidone, the hypoglycemia resolved, but symptoms of diabetes emerged. Therefore, it appears that the hypoglycemia induced by paliperidone may mask symptoms of diabetes. Paliperidone may induce hypoglycemia by increasing insulin secretion. This report could help elucidate the relationship between atypical antipsychotics and glucose metabolism.

## 1. Introduction

Paliperidone, 9-OH-risperidone, is the primary active metabolite of the atypical antipsychotic risperidone. It is reported to act via similar, if not the same, pathways as risperidone. Paliperidone was approved by the FDA for schizophrenia in 2006 (2010 in Japan). Researchers have proposed that it exerts therapeutic activity in schizophrenia through antagonism of central dopamine type 2 (D2) and serotonin type 2 (5HT2A) receptors. Hence, it is referred to as a serotonin dopamine antagonist (SDA) [1]. It is also active as an antagonist at *α*_2_-adrenergic and H1 histaminergic receptors. The various side effects of paliperidone include hyperprolactinemia, extrapyramidal symptoms, drowsiness, weight gain, sexual dysfunction, and hyperglycemia. While many studies have reported that atypical antipsychotics cause hyperglycemia, the link between hypoglycemia and antipsychotics such as paliperidone has not been extensively studied [2, 3].

## 2. Case Report

A 41-year-old unemployed, single woman with suspected insulinoma and a 13-year history of refractory schizophrenia (diagnosed according to The Diagnostic and Statistical Manual of Mental Disorders IV Criteria) was transferred from a mental hospital to the inpatient psychiatric unit of the Osaka General Medical Center (OGMC) for persistent hypoglycemia (fasting plasma glucose = 50~60 mg/dL) combined with elevated levels of immunoreactive insulin (IRI; 12.7 *μ*U/mL) and C-peptide immunoreactivity (CPR; 4.1 ng/mL). Although the patient did not present with any relevant physical or mental symptoms, she had previously been hospitalized five times for aggravation of psychotic symptoms. On this admission, she received antipsychotics (paliperidone 12 mg/day, bromperidol 36 mg/day, and levomepromazine 200 mg/day), mood stabilizers (sodium valproate 1200 mg/day), and benzodiazepines (flunitrazepam 2 mg/day and brotizolam 0.5 mg/day). While her psychiatric condition did not improve, side effects such as extrapyramidal symptoms and persistent hypoglycemia were observed.

Blood tests for insulinoma were requested, with the following results: fasting plasma glucose (FPG) = 51 mg/dL and immunoreactive insulin (IRI) = 12.7 *μ*U/mL. The results of clinical diagnostic tests for insulinoma were as follows: Fajans index (IRI/PG; PG: plasma glucose) = 0.24 (insulinoma > 0.3), Grunt index (PG/IRI) = 4.01 (insulinoma < 2.5), and Turner index (IRI × 100/PG-30) = 60.4 (insulinoma > 50). Her blood test results did not meet the criteria for insulinoma under the Fajans and Grunt indexes. Chest and abdominal computerized tomography (CT) did not reveal tumor lesions. Therefore, we suspected drug-induced hypoglycemia instead of insulinoma and promptly discontinued the benzodiazepines. However, we resumed oral administration of benzodiazepines when the symptoms of hypoglycemia did not resolve. We then reduced the dose of paliperidone, after which the symptoms of hypoglycemia gradually improved (Table 1). Although some of the patient's other medications could affect blood glucose levels (e.g., bromperidol and levomepromazine), they were not changed.

Diabetes management therapy, including blood glucose monitoring and diet therapy, was initiated after discontinuation of paliperidone because we detected glycophilia. Since the hyperglycemia persisted, we performed an oral glucose tolerance test (OGTT) to evaluate for abnormal glucose tolerance, and the following results were obtained: fasting: 61 mg/dL; 30 min: 204 mg/dL; 60 min: 172 mg/dL; 90 min: 64 mg/dL; and 120 min: 77 mg/dL. She was diagnosed with reactive hypoglycemia in the early stage of diabetes.

## 3. Discussion

Atypical antipsychotics markedly affect glucose metabolism, increasing the risk of incident diabetes [4]. While several cases of hypoglycemia caused by atypical antipsychotics have been reported, the mechanism is not completely understood. Suzuki et al. [5] found that risperidone, olanzapine, and quetiapine induce hypoglycemia by increasing insulin secretion in nondiabetic patients with schizophrenia. In their report, reducing risperidone doses ameliorated risperidone-induced hypoglycemia. In our study, discontinuing benzodiazepines known to cause hypoglycemia [6] did not alleviate our patient's hypoglycemia, but reducing the dose of paliperidone (an active metabolite of risperidone) effectively resolved her persistent hypoglycemia by dampening insulin release. However, symptoms of diabetes subsequently appeared.

Test results indicate that paliperidone induced hypoglycemia by increasing insulin secretion. We hypothesize that paliperidone-induced hypoglycemia may have masked the symptoms of diabetes that were present before the patient began taking paliperidone. There are several hypotheses on how atypical antipsychotics induce hypoglycemia. The secretion of insulin by pancreatic beta cells might be enhanced by atypical antipsychotics and consequently cause hypoglycemia [7]. Atypical antipsychotics might antagonize muscarinic receptors so that insulin secretion continues after glucose levels return to normal, leading to hypoglycemia [8]. Some studies also reported that risperidone significantly reduces glucose levels through induction of insulin release, but the effects might be caused by its potent antagonistic activity against *α*_2_-adrenoceptors [9, 10]. Paliperidone, the primary active metabolite of risperidone, has no affinity for muscarinic receptors. However, it does have affinity for *α*_2_-adrenoceptors (*K*_*i*_ of 3.9 nM) that is slightly stronger than that of risperidone (*K*_*i*_ of 151 nM). Hypersecretion of insulin in this patient might have been caused by inhibition of *α*_2_-adrenoceptors by paliperidone [11].

Because symptoms of early diabetes were observed after our patient discontinued paliperidone, we believe that paliperidone-induced insulin secretion may be associated with glucose metabolism in type 2 diabetes. For this reason, we suggest that clinicians monitor baseline blood glucose levels and perform OGTTs before initiating paliperidone therapy in high-risk patients with type 2 diabetes and type 2 diabetes with residual insulin secretion.

## Figures and Tables

**Table 1:** Dose-dependent changes in glucose, fasting insulin level, and C-peptide immunoreactivity.

Paliperidone (mg/day)	Glucose (mg/dL)	Fasting insulin (*μ*U/mL)	C-peptide immunoreactivity (ng/mL)
12	51	12.7	2.38
6	66	8.4	2.18
0	67	4.4	1.39
